# Digitizing Social Counseling—Insights for Workplace Health Management

**DOI:** 10.3390/ijerph19020917

**Published:** 2022-01-14

**Authors:** Wiebke Schlenger, Marlies Jöllenbeck, Tjorven Stamer, Angelika Grosse, Elke Ochsmann

**Affiliations:** 1 Institute of Occupational Medicine, Prevention and Workplace Health Management, Medical Faculty, University of Luebeck, 23562 Luebeck, Germany; marlies.joellenbeck@uksh.de (M.J.); tjorven.stamer@uksh.de (T.S.); elke.ochsmann@uni-luebeck.de (E.O.); 2 Employer’s Liability Insurance Association for Health Services and Welfare Care (BGW), 76137 Karlsruhe, Germany; angelika.grosse@bgw-online.de

**Keywords:** digital transformation, social counseling, mail counseling, stress and strain, workplace health management

## Abstract

Working digitally can lead to changes in work organization and social interactions, as well as work pace and workload. Online counseling is more and more integrated in social counseling. Research exists on employees’ and users’ attitudes towards online counseling as well as on the advantages and disadvantages of online counseling. There is a lack of studies on the stressors and strains caused by the increasing digitalization and the associated health consequences in this context. With an interview study, we investigated the general work situation of counselors, with a focus on stressors, strain, and resources caused by online counseling. Consecutively, we discuss the results in relation to their impact on workplace health management. Twenty-two explorative interviews with counselors from a German welfare organization were conducted in 2019 and 2020. Qualitative content analysis according to Mayring was used for analysis. Counselors’ use of online devices depends on their own digital affinity and is likely to be used when advantages for clients are seen. Difficulties were mentioned in establishing a relationship of trust with the clients. Good teamwork and regular informal exchanges among colleagues contribute to job satisfaction. Overall, we found only few health-related effects. Results of the study suggest that digitization can have positive effects on the job satisfaction of counselors, if the associated changes are supported by organizational measures.

## 1. Introduction

The use of online consulting services has been steadily increasing for several years [1,2]. This development toward digitalized social work is gaining momentum, and not just as a result of the COVID-19 pandemic [3,4]. It results in changes in the general work situation of counselors (also referred to as “social consultants”, “consultants”, or “social counselors”), challenges for their occupational health management, and changes in the perception and implementation of psychosocial counseling. Digitization and mobile work can thus also have an impact on the health situation as well as on stress and strain patterns of providers of social counseling and psychotherapy.

### 1.1. Definition of Online Counseling

Online counseling is defined variously within the literature. It is generally described as counseling by means of computer-based communication carried out by a professional counselor or psychologist using various media such as e-mail, chat, or video systems. The communication can be synchronous or asynchronous and may occur alone or in combination with face-to-face counseling [5,6].

In this article we use Mallen and Vogel’s (2005) definition of online counseling and therapy:

“*Any delivery of mental and behavioral health services, including but not limited to therapy, consultation, and psychoeducation, by a licensed practitioner to a client in a non-face-to-face setting through distance communication technologies such as the telephone, asynchronous e-mail, synchronous chat, and videoconferencing.*”

[7]

### 1.2. Impact of Digitalization on Work

The increasing digitization of the workplace can have far-reaching consequences for employees. In particular, studies are available on the benefits and risks in the working world of the so-called Industry 4.0. Working digitally, often addressed as “new work”, can lead to changes in work content, organization, and environment, as well as social interactions at the workplace [8]. This development not only leads to less loyalty but also to an increased work pace and workload [8,9].

Studies show that information and communication technologies (ICT), depending on how they are implemented, can lead to an increase or decrease in productivity, performance, and job satisfaction [10]. Increasing work flexibility can contribute to a healthy work–life balance, as virtual employees benefit from fewer distractions, a more pleasant work environment, and the ability to work outside of regular hours [11]. Thus, workplace virtualization can also be beneficial for employees with special needs, caregiver relationships or other commitments.

However, working digitally also poses risks to employee health. One of the consequences of digitalization is technostress, which leads to employees working more and faster with many different, rapidly changing technologies. This can lead to information overload and social isolation, as well as an increased perception of stress, which in turn can lead to health risks such as burnout and depression [12,13,14].

Changes are also caused in teamwork between virtually working teams. In face-to-face communication, gestures and facial expressions can contribute to a different understanding of what is being said than in online communication [15]. It has been shown that employees in virtual teams also need personal contact in order to establish a basis of trust and a bond with their employer [16].

Thus, challenges for workplace health management arise out of “new work” [17]. Automated processes and human–machine communication make it necessary to acquire extended specialist knowledge, for which training courses are often offered [18]. The possibility of digital communication between employees and supervisors should lead to improved collaboration and thus to an increase in job satisfaction [18]. Approaches that address, for example, the increased monitoring of employees through the collection of data within processes or the flexibilization of work are still pending [19].

The transferability of these activities to the field of social work is unclear. An evaluation of occupational health management for social professions in the digital transformation is still lacking to a large extent.

### 1.3. Stress and Strain Patterns in Social Work

Social work employees have a diverse work profile. Their work focus is on counseling clients with complex social and mental issues. They are also often involved in providing workshops and training for socially or mentally impaired people. In addition, a large part of their working time falls on documentation and research [20]. Because of the demanding work of social counselors and psychologists, this group is also at special focus when it comes to health effects of work such as burnout and exhaustion [21,22,23].

Identified factors that lead to an increased possibility for adverse health effects are mainly found on the organizational level. A high workload, working overtime, financial hurdles as well as a lack of scope for action all contribute to the perception of stress [22,24,25]. Working with complex problems or aggressive clients, can also cause distress [23]. In addition, lack of staff resources and long waiting lists for counseling appointments can be among the stressful factors [24]. Counselors also experience isolation when not working as part of a team [26].

The consequences of these occupational risk factors can include physical and psychological health problems, such as burnout, emotional strain, exhaustion, and cardiovascular problems, among counselors [27,28]. Burnout can also lead to a decrease in work engagement, job satisfaction, and quality expectations [22,29].

Studies have identified factors that reduce the risk of illness among consultants. Personal resources, such as opportunities for self-reflection, self-care, and a good work–life balance, can reduce strain [23,25]. Organizational factors, such as good managerial support and adherence to work schedules, can also contribute to reduced risk [20,22,24].

The increasing digitization of the workplace in social counseling represents a new variable in the description of stress and strain. Studies on the impact of digitalization on health in the field of online counseling are currently rare.

### 1.4. Counselors’ Perceptions of Online Counseling

At present, some results are already available on how consultants perceive online counseling and assess its advantages and disadvantages.

#### 1.4.1. Therapeutic Alliance

The biggest concern about online counseling on the part of counselors is the difficulty of establishing a basis of trust and a therapeutic relationship. In several studies, counselors stated that they felt more secure in establishing a relationship in face-to-face counseling than in an online setting [2,30]. This confidence to establish a therapeutic relationship in face-to-face counseling is also related to the experience of the individual counselor, whereas the ability to establish a therapeutic relationship in the online setting does not necessarily correlate with the experience of the counselor [2].

A good therapeutic relationship relies on agreeing on common goals and tasks and building a relationship of trust between the participants [31]. In online counseling, telepresence also plays a role for this purpose, which can lead to achieving the goals and building a strong relationship with the client [32].

Reasons for perceived difficulties in relationship building can be manifold. Counselors state that they feel that the timing of the counseling process is different from face-to-face counseling. More time is needed to gather the necessary information and move forward with relationship building, so that creating and achieving goals take a lesser role [33,34,35]. An often-abbreviated counseling process also causes uncertainty. Consultation contacts often take place over only a short period of time [36]. Nevertheless, initial results show comparable effectiveness. In addition, some studies indicate that the establishment of a therapeutic relationship can be strengthened because clients reach the core of the problem more quickly due to perceived safety and anonymity, thus speeding up the counseling process [37].

#### 1.4.2. Absence of Gestures and Facial Expressions

Text-based counseling leads to an absence of nonverbal cues, facial expressions, gestures such as hand movements, and voice color [33,37,38]. This leads to the criticism that misunderstandings and miscommunication may occur more frequently during counseling [30,37,39]. Furthermore, counseling in online settings can be complicated by the difficulty of conveying necessary emotions, empathy, and understanding in asynchronous text messages [6,40].

Consequently, it can be deduced that both, the counselors and the clients, must be able to express themselves well in text [37]. In a qualitative study by Mishna et al., social work interns who compared conducting online counseling and face-to-face counseling within a study indicated that it was more difficult for them to respond emotionally to clients’ problems due to the lack of nonverbal communication. Transferring relationship building and compassion skills from face-to-face counseling to text-based online counseling can also be difficult [30].

#### 1.4.3. Anonymity

In addition to the altered therapeutic relationship, a disadvantage and uncertainty for the counselors and therapists can lie in the anonymity of the clients. Even if this circumstance can lead to an easier information situation, the anonymity means less access to the clients in particularly difficult situations, such as sexual abuse, domestic violence, or threat to the life of themselves or others [39,41].

#### 1.4.4. Security and Technical Difficulties

The use of new technologies in social counseling includes advantages for counselors, as notes on the counseling process, information gathering as well as storage of data are facilitated. It also offers advantages when trainees can ask direct questions and the consultation texts can be edited together [42]. A user-friendly interface with privacy standards or additional applications such as whiteboard use or group rooms are considered useful [38].

However, difficulties with technology, such as a slow and unstable Internet connection and inadequate technical equipment on the side of the counselor or the client, can stand in the way of a satisfactory consultation process [38,39,43].

### 1.5. Theoretical Models of Our Qualitative Study

#### 1.5.1. Job Demands Resources Model

The Job Demands Resources Model by Demerouti et al. conveys the impact of job demands and resources on health and work engagement.

Demands in this context include factors such as time pressure and poor team cohesion. Resources include latitude, job security, and support from colleagues and supervisors. Interactions exist, as work resources can reduce the negative effect of high work demands and, at the same time, high work demands can reduce the positive effects of resources [44].

#### 1.5.2. Stress and Strain Model

The stress–strain model of Rohmert and Rutenfranz shows the interrelation of these factors. Stress is understood as external factors such as time pressure, inadequate ergonomics of the workplace and lack of support from managers. Personal resources lead to a different perception of these factors. The interaction of individual conditions and stresses leads to different levels of strain. This can be positive and thus encourage and motivate employees in their behavior, leading to increased job satisfaction and well-being. However, the immediate effects of the stress can also be perceived as negative in the case of other personal resources and then lead to psychosomatic disorders, fatigue, and burnout [45].

#### 1.5.3. German Guideline of (Mental) Health and Safety (GDA)

The risk assessment in companies provides a basis for occupational health and safety action.

The German Occupational Health and Safety Act requires the performance of the systematic risk assessment, the derivation of suitable measures and appropriate documentation. The requirements are divided into five fields of action, namely work content, work organization, work environment, social interaction, and flexibilization/mobile work [46].

### 1.6. Study Objective

The increasing digitization of social counseling has been starting since before the start of the COVID-19 pandemic.

Various studies on the impact of digital mental health services on clients and counselors have been conducted and indicate advantages and disadvantages as well as effects on the work environment. However, although most studies focus on effectiveness and user perception of online offers, little research exists on the general working conditions of social workers, the health impact of the digital transformation as well as the challenges for workplace health management. As it stands, there is little evidence on the stress and strain patterns and health effects of digitized counseling formats among those who use them professionally.

By means of the interview study the field of action of health-related risks, stress and strain in social counseling is explored. The interview study sheds light on the work situation regarding work content, organization, and environment as well as social interaction and work flexibility. In addition, the work context is discussed regarding the demands and health-related effects of the digital transformation and the associated stresses and strains. The study aims to also highlight fields of action in workplace health management regarding the working conditions during the digital transformation in social counseling.

## 2. Materials and Methods

The following section is guided by the consolidated criteria for reporting qualitative research (COREQ) and the standards for reporting qualitative research [47,48].

### 2.1. Study Design and Setting

This qualitative interview study utilized semi-structured interviews conducted with 22 social consultants associated with a German welfare organization. The method was used to explore the action fields of the risk assessment of digital transformation in the consulting setting. The interviewees operate throughout Germany in different counseling fields (e.g., addiction, pregnancy) and use a specialized consulting software that is gradually implemented at all consulting locations of the organization.

Data protection as well as ethical and scientific standards in line with the declaration of Helsinki have been completely considered. A positive vote of the ethics committee of the University of Luebeck is on hand. Participation in the interviews was voluntary.

### 2.2. Participant Recruitment

The respondents were recruited in an internal procedure by means of a call via the networks of the faith-based welfare umbrella association. Care was taken to ensure a mix of the various counseling fields (e.g., addiction, pregnancy, suicide) and group affiliations. Sufficient saturation was achieved, as no more new findings could be achieved in the subject area under investigation.

### 2.3. Data Collection

A semi-structured interview guide was developed according to Mayring’s social science quality criteria for qualitative interview studies [49]. The questions focused on the five fields of action of the German guideline of (mental) health and safety [46]. The methodological alignment based on the Job Demands—Resources Model by Demerouti et al. and the Stress and Strain Model by Rohmert and Rutenfranz, described earlier [44,45].

The interview guideline included questions on work content, work organization, the working environment, social interactions, and mobile work. In addition, questions were asked about the assessment of quantitative and qualitative strains and demands, as well as reasons for job satisfaction. Furthermore, participants were asked to give an assessment of various aspects of online counseling, focusing on advantages, disadvantages, and software ergonomics of the digital consulting setting. The questionnaire was presented to in-house experts in order to appropriately bind specific aspects and the wording of the setting to be studied. A pre-test was conducted with n = 6 to test comprehensibility, practicability, and manageability. Based on the results of the pre-test, the questionnaire was finalized.

Three interviewers were briefed according to existing quality standards for qualitative interviews and conducted the interviews on a pro-rata basis. Written informed consent was obtained prior to the interview. The interview partners were informed comprehensively about the aims and purpose of the study, anonymity, and data protection.

The interview partners were fully informed that they could stop the interview at any time without giving a reason. The interviews were conducted between October 2019 and February 2020 via telephone and once face-to-face at the workplace.

All interviews were digitally recorded and lasted on average 50 min.

Subsequently, the interviews were transcribed and anonymized by trained staff members. The data were then transferred to the coding software in compliance with data protection standards.

### 2.4. Data Analysis

Between April 2020 and June 2020, the interviews were analyzed using a qualitative content analysis according to Mayring with the data and text analysis software MAX-QDA, version “Analytics Pro 2020” (VERBI GmbH, Berlin, Germany) [49]. The coding using a coding guideline was designed as a deductive analysis process along the interview guide and allowed new codes in the sense of inductive exploration of the field in order to maximize the gain of knowledge. Different methods to reach trustworthiness were used. Firstly, the method of investigator triangulation was used. Two scientific staff members carried out the coding for quality assurance of the procedural quality. The coders exchanged views frequently to discuss questions about quotes, codes, and interpretation. Secondly, an audit trail, in form of a shared document with access only for the researchers, was used.

## 3. Results

### 3.1. Sample

The participants were divided into four groups according to their online counselling experiences. For the exploration of the research field, it was necessary to take a closer look at different user groups within the organization in order to better understand possible systematic differences, e.g., with regard to the motivation to use online counseling and the effects of online counseling. Against this background, sub-groups were formed. The sample information is displayed in Table 1.

Participants in group one did not conduct online counselling but only face to face consulting. The average age in this group was 51.5 years with a company affiliation of 18 years. Four participants were female, and one was male.

Participants in group two conducted a little online counselling (less than two per month) and face to face consulting. The average age in this group was 47.0 years with a company affiliation of 18.5 years. Three participants were female, and two were male.

Participants in group three conducted online counselling often (at least once a week) as well as face to face consulting. The average age in this group was 58.5 years with a company affiliation of 38.5 years. Four participants were female, and one was male.

Participants in group four conducted online counselling with two different software programs; therefore, they could make statements about the usability and web interface of the program. The average age in this group was 29.6 years with a company affiliation of 5 years. Four participants were female, and three were male. Four of the participants were volunteer counselors in the U25 suicide prevention program, in which counselors are not allowed to exceed the age of 25 themselves.

All interviewees conducted counseling in social fields such as insolvency, pregnancy, and addiction counseling.

The following results are presented in order to the systematic structure of the German guideline of (mental) health and safety (GDA).

### 3.2. Job Content

#### 3.2.1. Responsibilities

The main part of the day consists of the actual consulting work. Here, the focus is on face-to-face consulting. Online counseling is conducted with varying frequency, depending on the group surveyed. Further regular working time is spent on documentation and filling out applications. Depending on the field of counseling and location of the advice center, the documentation is either carried out digitally in a database, by means of in-house software, or, rarely, analog. Currently, there is no possibility to document online counseling directly digitally on the side. The consultants state a documentation time of around 30 min per consultation occasion.

Other duties may vary depending on location and consulting field. Some counselors do prevention work, for example in schools, others work in care or in other support services, such as workshops or so-called mothers’ cafés.

*Group 2: “After [the counseling session] comes all the processing. Applications to the regional foundation, help for mother and child, applications to the bishop’s ore fund, the archbishop’s aid fund, that’s what it’s called. Everything that is brought up in the counseling sessions has to be processed. Collection of data, statistics. (…) Yes, that is the normal daily routine. We always have consultations scheduled for one hour. Often there are follow-up consultations on other days. Then new appointments are made if the client wants them.”*

In addition, many counseling centers hold regular supervision sessions, in which the consultants regularly participate.

*Group 3: “We have intervision on a regular basis and we also have supervision every now and then. So that is important. Currently, we have group visions four to six times a year. There are different people from other centers, but from the same institution. I find that very beneficial.”*

#### 3.2.2. Design of the Consulting Activity

Online counseling is currently used mainly as mail and chat counseling by respondents. A company software program is used for this purpose. The willingness to use online consulting depends on the digital affinity of the consultants rather than the age. When benefits are seen for clients, counselors are more willing to offer online counseling. However, the longer they work online, the more critical the respondents are of advancing digitization. Group 1 and 2 state more frequently than the groups 3 and 4 that expanded digital offerings (e.g., online supervision, online team building, online group consultations) are desired and imaginable.

*Group 4: “At the same time, I think there could also be risks, for example, in outsourcing all therapy activities to the digital realm and saying: we’re now only doing digital things. So especially, for example, in a long-term therapy support or in outpatient treatment, I could hardly imagine doing the whole thing simply as an e-mail consultation or even as a webcam session or something.”*

Across all groups, disadvantages for counselors and advantages for clients are mentioned frequently. Client-related benefits and progress achieved through online counseling are frequently cited throughout the interviewees. The spatial and temporal flexibility for clients is mentioned as positive, as well as the low-threshold access and the anonymity of the client. Counsellors prefer to use online counseling for initial interviews before they offer a face-to-face meeting to their clients. In particular, interviewees with less work experience with online counseling state that it is better for clarifying procedural matters rather than actual counseling sessions.

*Group 4: “They have the option to remain anonymous. They have the possibility to end the contact even suddenly, without ever having to apologize or justify themselves. You don’t have to make any appointments. So, all in all, I think this is simply a low threshold offer that also accommodates the clients through all this flexibility. ”*

Online obstacles for the consultants are especially the anonymity and the non-binding nature. On the one hand, the lack of facial expressions, gestures, and voice recognition makes it difficult to establish a basis of trust. On the other hand, counselors with more experience in online counseling state that clients gain trust more quickly and that the counseling process is simplified by opening up to counselors early on.

Counselors who do not yet use online counseling fear misunderstandings due to the written format but are willing to establish online counselling to meet the needs of clients.

*Group 2: “So you have no facial expressions, no gestures, you don’t hear the voice. You have to trust that what the person writes, is true. (...) Most of the time, I only have one contact in online counseling, so building a relationship is almost an exaggeration; it’s more like conveying information. Of course, there are also longer conversations. I have chatted back and forth for an hour or an hour and a half. That’s more intensive, and you also get closer to the people. But that’s about it.”*

Group 4 was asked to evaluate the software ergonomics separately. Interviewees state that thanks to the software update, the user interface is more appealing, and the program can be used more efficiently. However, the chat layout of the program is criticized. According to the interviewees, this encourages clients to write only short messages, with which the development of the counseling process is only possible slowly. Occasional technical difficulties and the lack of a read confirmation also pose problems.

*Group 4: “But the new system now also targets the chat. I see this as an extreme disadvantage for an e-mail consultation. When someone registers with us and sees this, they think they will get an answer immediately. That means we often get very short initial emails and also the complaint: why doesn’t anyone answer me here? Well, because we are not a chat consulting service.”*

#### 3.2.3. Scope of Action

Respondents indicate that the workday cannot be planned entirely due to frequent unforeseen events, such as emergency consultations, complex problems, or non-appearance of clients. However, unlike face-to-face counseling, online counselors are more likely to adjust to short counseling occasions, sudden breakoffs of conversations, and less obligation.

Nevertheless, it is important for the consultants to be able to plan the day independently as far as possible and to have defined time periods for appointment consultations, emergency consultations, and online consultations, considering time for documentation and organizational work.

#### 3.2.4. Emotional Involvement

Most interviewees report that they are able to distance themselves well from their clients’ problems. Nevertheless, mail consultation can lead to mail being answered after work hours. However, the interviewees state that they do not yet feel burdened by this.

However, social counseling can also be emotionally demanding for counselors. Across all groups, the statement is made that the counselors feel stressed when client expectations cannot be met due to financial, bureaucratic, or time hurdles. If one’s own quality standards regarding the counseling process cannot be met, this represents a burden.

*Group 3: “So as I said, if the woman is once again on the brink of the edge or was beaten, but still gets back again. Or the child has died. In so stressful situations, I think to myself: Yes, it’s bad, but it’s not mine and I leave it here. And then I put the file in the cabinet, lock it up or just lock up the office or leave it, but sometimes you do think about it at home.”*

Counselors in face-to-face counseling indicate that especially verbally aggressive individuals can be emotionally demanding.

Online counselors, in contrast, cite emotional distress when they feel that mail counseling reaches its limits, and they can no longer reach clients through written words. In addition, the non-committal nature of the counseling process poses a particular challenge, as clients often break off the counseling session for no apparent reason.

When asked, respondents negate health effects such as burnout or exhaustion symptoms. They state that they feel healthy and have no physical complaints related to work. Respondents across all groups report not noticing any health effects from stress at work and not having any mental or physical problems.

*Group 4: “I am usually relatively satisfied with the quality of the counseling. But of course, there are moments when I wish I could do more [...] But that is again due to the medium of mail, I would say. Sometimes you sit there a bit helplessly and would like to do more than you can.”*

### 3.3. Work Organization

#### 3.3.1. Working Time

In addition to the low predictability of the workday, respondents describe a high workload in general. The counselors interviewed reported that they have to work overtime to meet their clients’ needs and thus meet their own quality standards. The high demand for consultations also creates the feeling of being under time pressure while working. This can result in the consultants’ own quality standards not being fulfilled, thus leading to a reduction in job satisfaction. In particular, additional tasks such as workshops, training, or evening events can lead to overtime and affect the consultants’ time schedule. Additionally, when colleagues are absent due to illness or vacation, working hours can easily be exceeded.

*Group 3: “When people [clients] are completely unnerved and under a lot of pressure, they pass it on. That is difficult for the administrators, and it is also difficult for us. And sometimes women like me get carried away and take on too much. So, it’s certainly not a must according to the rules, but it’s also more the pressure that comes from many sides, I’d say.”*

However, the respondents state that the number of overtime hours is kept within limits or can be reduced at times. Thus, working overtime mostly does not feel like a burden.

In addition, interviewees in groups 3 and 4 state that it is important to have defined time periods for online counseling. Mail and chat consultations cannot take place in between. For online consultations, specific time frames are required to respond with a well thought out message to meet their own quality standards for consultation.

Respondents who do not yet participate in online counseling indicate that it is not clear how online counseling will be implemented into their day.

*Group 3: “But there is a lot of uncertainty and a lot of fear at the moment with the new system. It has been planned and developed for a long time, has dragged on forever, and now all of a sudden, it’s starting. I can’t do it, I don’t want to do it, it overwhelms me, between the daily consulting routine, there was a lot of anxiety at first. It’s not really possible to do that on the side.”*

#### 3.3.2. Communication

Both digital and analog forms of communication are used. Among colleagues in a department, communication is often face-to-face. In particular, brief consultations take place in analog form. Some teams also use messengers for brief information purposes. In general, most counselors state communication among colleagues as uncomplicated.

*Group 1: “For me, it’s really really old school, I would say, which means that I talk to my colleagues here directly or I talk to them on the phone when she’s in the field office. Or I just write an e-mail. That’s no way to talk about, well, never about client data. That’s just organizational stuff. But when it comes to clients, when it comes to content, then really only in direct exchange or on the phone.”*

With superiors and other departments, communication is generally digitally via e-mail. Technical information that affects many employees is also shared in this way. However, if regular team meetings with superiors and sometimes external cooperation partners exist, they mostly take place in person.

### 3.4. Work Environment

The ergonomics of the workplace are described as predominantly good. The employers also attach importance to the right equipment with office materials, lighting conditions and technical devices. The respondents’ wishes for office equipment are often fulfilled. However, technical difficulties such as unstable internet connection or problems with software updates are described, especially in group one of the respondents.

*Group 3: “We have very nice rooms. It is also prescribed that they must be soundproof. We can design them ourselves; we have new office furniture; the rooms are bright and quiet. Yes, we have a consulting desk, it is necessary that we have our area within the office and a waiting room for the clients.”*

### 3.5. Social Relations

#### 3.5.1. Among Colleagues

The social interaction between colleagues is described by the interviewees as good to very good. Counselors see informal sharing and support for challenging counseling occasions as an important resource. The exchange takes place among all respondents in presence. Online supervision or team meetings are not well envisioned by the respondents. The interviewees also see the long-standing teams and the resulting good teamwork as a resource.

*Group 2: “We have a really, really great team where we can all rely on each other. And if one of us somehow has a problem and is at his limits or has any issues where one doesn’t really know how to deal with them, there’s always someone who has an open ear. It goes without saying that we all look after each other when we have the feeling that someone is not doing well.”*

#### 3.5.2. Towards Supervisors

The respondents state that the cooperation and relationship with their respective supervisor can be described as good. Leaders are perceived as supportive when work-related problems arise and offer and support training for their employees.

*Group 3: “So I find [my boss] very supportive when I have personal problems, very good back-up. I also find her very supportive when it comes to professional development. My boss is, of course, an incredibly committed person in all kinds of areas. So sometimes it’s a bit difficult when someone is overworked.”*

Few interviewees indicate that there are significant problems with their supervisor and that they find themselves poorly supported and valued. If the manager is burdened by external circumstances such as high sick leave or organizational changes, the relationship with the employees suffers.

*Group 1: “On the whole it’s good. I feel that the workload of my manager, that is, my department head, has increased so massively in recent years and continues to do so. […] If the workload is at a good level, then it’s great, the collaboration with her is really good and profitable and supportive and yes, also instructive in many places. And if the workload is just too heavy, at least that’s how I assess it, then it becomes a burden. At least for me. Then it’s rather the case that the climate is very bad for me.”*

### 3.6. Flexible Working Conditions

Only a few of the respondents presently use the option of working from home. There is currently a lack of clear guidelines for flexible use of working time when it comes to working from home. In particular, regulations on the recording of working hours and data protection are not familiar to the respondents but are considered important.

Interviewees that do work from home often use this possibility for carrying out organizational work.

Conceivable advantages for the interviewees could be flexibility in terms of time, the elimination of long commutes and the tranquility of the private environment. If the consultants do not yet use the option of working from home, they cite their current life situation as well as the fear of not being able to distance themselves from the consulting process as reasons for not using it.

If the interviewees consider counseling from the home office, it does not depend on the degree of digitization of their work but rather on the understanding that they are only able to distance themselves well when counseling takes place in their office.

*Group 3: “But that certainly also has to do with the life situation. As my child was smaller or as I also experience with other counselors, it can be helpful to say, I don’t have time in the afternoon anyway. And then I would log on to the chat again in the evening for two hours. That can actually be a working model that is helpful for employees. But I also believe that it requires very strong security and a set of rules about how the data is protected. Can someone read it? What do you do with written notes? Are the files stored securely? And also, for self-protection, do I get the full times written down?”*

## 4. Discussion

The aim of this study was to shed light on the work situation of social counselors in the context of the digital transformation process and to identify stressors, strains, health effects, and challenges for workplace health management that can arise from digitizing social counseling.

The starting points for prevention present themselves as very diverse. However, no changes in the work content were identified as a result of online consulting. Digital consulting is primarily understood by the respondents as a supplementary tool and accordingly not perceived as an additional task. Nevertheless, this perception might have changed during COVID-19-related lockdown periods. Thus, the online counseling service should be well integrated into the daily work routine. It must be precisely scheduled and timed so that it is not perceived as a burden. The findings indicate that organizational framework conditions must be created for the successful and health-oriented implementation of online counseling in the working day, although this decreases the individual degree of freedom. In addition, results show that possibilities for communication with and support from colleagues and supervisors seem necessary to be able to cope with the introduction of online consulting.

Whether online consulting is viewed positively in this study does not depend on age, but rather on the technical affinity and the experience of the consultants, whereas other research found that the certainty of being able to establish a therapeutic relationship is not dependent on the experience of the counselors in E-therapy [2]. We conclude that, as long as the use of online counseling is a free choice, technical affinity is the main factor in perceiving the advantages of online counseling, and an understanding of technology and the willingness to use it are key factors in creating a positive experience.

It has been shown that it causes emotional stress when counselors feel they cannot adequately help their clients due to financial and time constraints [22,24,25]. Dealing with aggressive and stressed clients is also a challenge [23]. Here, online counseling can be a useful supplement and alternative since the physical distance also creates a barrier against physical aggression as well as an emotional demarcation. Therefore, it might be especially helpful for counseling areas with potentially aggressive clientele.

However, the virtual distance between counselor and client does not only offer advantages, but can also reduce the perception of helpfulness of the treatment. As can be found in the literature, the limited possibility of transmitting empathy and emotions with online counseling and the lack of perception of gestures and facial expressions are particularly perceived as impairing [33,37,38]. This can lead to consultants seeing their own quality expectations of their work as insufficiently met. Video counselling might help in these situations.

According to the interviewees, job satisfaction is particularly reduced if clients cannot be counselled according to their own quality standards, despite the effort they are giving. In addition, the results also indicate that perceived time pressure, bureaucratic hurdles, and overtime are burdensome [22,24,25].

In previous studies, counselors consistently indicated that technical problems were a major hurdle in the smooth running of online counseling [38,39,43]. In this study, consultants also indicated that software and Internet problems are an obstacle to guarantee satisfactory online counseling services and job satisfaction. Here, too, technical affinity and training represent an important resource for shaping the digital transformation in a way that is compatible with health. Organizational software should be designed in such a manner that it can be used intuitively and has features that advance the consultation process and bring advantages compared with face-to-face consultation. It will then be perceived as positive and the will to use online counseling programs increases. Additionally, organizations minimizing expenditures for technical support are probably saving in the wrong place, especially in a digital change process.

Further, the digitalization of the working world is changing communication as teams increasingly work virtually and digital communication is encouraged [15]. Respondents also cite increasing digitization in this area. However, according to our results, the greatest resource for the counselors is the informal, personal exchange and collaboration in the team on site and face to face. Working exclusively from home could pose problems in terms of the health-related design of the work. In addition, the counselors report that they value conducting the consultation from the office in order to be able to establish a better work–life balance and to better distance themselves from the problems and issues of their clients. In the case of teamwork, they also value a long-term working relationship and thus a relationship of trust between the employees. This is in line with the results from other studies, which indicate isolation of counselors as a risk factor for burnout and emotional exhaustion [26], and means that even in times of a massive increase in digital work options, personal interaction can prove beneficial for employee health and times and places should be planned for in organizations.

In the welfare organization, mobile work did not yet play a major role at the time of the survey and was used more for administrative tasks. However, it is recognized by the interviewees that the possible isolation, the lack of informal communication and the need for changed demarcation mechanisms, could represent risks for the healthful design of work. Nevertheless, it can be an advantage for certain groups of employees. Here, it is necessary for organizations to develop individual guidelines and new health offerings that can make it easier for these employees to work from home.

The results are consistent with the theoretical models used. Strains such as time or technical hurdles, emotional involvement in clients’ problems, and problems reaching clients through online counseling led to feelings of being stressed. If personal resources, in this case informal exchange between colleagues, spatial separation as well as organizational support, are available, they can reduce negative health effects and increase job satisfaction despite stress. More than ever, one aspect of digital work can pose a source of stress for one employee, whereas it is seen as resource from another. The changing work calls for more individualized concepts of prevention and health promotion in highly qualified workforces.

## 5. Conclusions

In conclusion, this study outlines central points for a health-oriented design of work and workplace health management in an online setting of psychosocial counseling.

Firstly, counselors should be able to decide to a certain extent for themselves whether to offer online counseling, since voluntariness and technical affinity positively influence willingness and implementation. In addition, it is also necessary to consider that online consulting, if used, has to be integrated into the daily work routine. Especially in the early hours of these changes, time resources have to be calculated. Employers should cooperate with employees in this sense and jointly design the working hours and daily work routine.

Secondly, if the working environment, namely, the technical equipment, technical support, technical education, as well as a good team cohesion, can be guaranteed, health-oriented work can likely be ensured. Possibilities for an exchange between consulting events with other counselors about work problems should also be guaranteed within the framework of the confidentiality obligation. For this purpose, organizational measures should focus on the above-mentioned aspects, while including employees in the design of these measures (bottom-up).

Thirdly, another challenge in the conversion of counseling services to an online setting is the increasing flexibility and mobile working. The most important resources mentioned by the respondents relate to the convenience of working from the office or working as a part of an on-site team. This should be taken into consideration when introducing counseling from the home office and thus less intramural face-to-face interaction between clients, colleagues, and superiors.

As delineated in this study, the fields of action for occupational health management in the digital transformation in the social sector include, above all, the restructuring of work organization and social relationships. Digitization and the new requirements that arise should be included in a risk analysis that can help to develop more individualized interventions for the workplace health management. Additionally, they should be able to consider life phases, like child-upbringing, into a holistic plan for workplace health management. This study shows possible courses of action for occupational health management and should be included in the revision of existing concepts for occupational health. In addition, it can be a starting point for reviewing new measures within occupational health management.

## 6. Limitations

Like any study, ours has limitations. First, the study involved a small sample of consultants from only one company, so generalizing the results may have weaknesses. The sample presented itself as particularly healthy, this contradicts results of other studies that surveyed a larger collective of social consultants. Further research with a larger and more widespread sample would be helpful. Furthermore, driven by the COVID-19 pandemic, mobile working and home offices have become more prevalent. This development should be taken more into account in further studies, researching risk analysis for social counselors and developing standardized questionnaires to better assess health-related stressors and strains posed by the digital transformation in counseling. In addition, workplace health management involving online counseling and digitized workplaces should be investigated in further studies. In concrete terms, this exploration of the research field represented for the authors the starting point for an extensive, quantitative research project with the development and application of a quantitative questionnaire. This should be used as a starting point for the health-oriented further development of the digital transformation and the working conditions in the online consulting sector.

## Figures and Tables

**Table 1:** Sample Information.

	Group 1 (n = 5)	Group 2 (n = 5)	Group 3 (n = 5)	Group 4 (n = 7)
Age in Years	51.5	47.0	58.5	29.6
Sex Female:Male	4:1	3:2	4:1	4:3
Company Affiliation in Years	18	18.5	38.5	5

(Group 1: no online counseling; Group 2: little online counseling; Group 3: online counseling often; Group 4: online counseling with different software systems).

## Data Availability

The data that support the findings of this study are available from the corresponding author, [WS], upon reasonable request.
